# Alloimmune Risk Stratification for Kidney Transplant Rejection

**DOI:** 10.3389/ti.2022.10138

**Published:** 2022-05-20

**Authors:** Oriol Bestard, Olivier Thaunat, Maria Irene Bellini, Georg A. Böhmig, Klemens Budde, Frans Claas, Lionel Couzi, Lucrezia Furian, Uwe Heemann, Nizam Mamode, Rainer Oberbauer, Liset Pengel, Stefan Schneeberger, Maarten Naesens

**Affiliations:** ^1^ Department of Nephrology and Kidney Transplantation, Vall d’Hebrón University Hospital, Barcelona, Spain; ^2^ Department of Transplantation, Nephrology, and Clinical Immunology, Edouard Herriot Hospital, Hospices Civils de Lyon, Lyon, France; ^3^ Department of Surgical Sciences, Sapienza University of Rome, Rome, Italy; ^4^ Department of Nephrology and Dialysis, Medical University of Vienna, Vienna, Austria; ^5^ Department of Nephrology and Medical Intensive Care, Charité Universitätsmedizin Berlin, Berlin, Germany; ^6^ Eurotransplant Reference Laboratory, Department of Immunology, Leiden University Medical Center, Leiden, Netherlands; ^7^ Department of Nephrology, Transplantation and Dialysis, Bordeaux University Hospital, Bordeaux, France; ^8^ Kidney and Pancreas Transplantation Unit, University of Padua, Padua, Italy; ^9^ Department of Nephrology, Technical University of Munich, Munich, Germany; ^10^ Department of Transplantation, Guy’s and St Thomas’ NHS Foundation Trust, London, United Kingdom; ^11^ Centre for Evidence in Transplantation, Nuffield Department of Surgical Sciences, University of Oxford, Oxford, United Kingdom; ^12^ Department of General, Transplant, and Thoracic Surgery, Medical University of Innsbruck, Innsbruck, Austria; ^13^ Department of Microbiology, Immunology, and Transplantation, KU Leuven, Leuven, Belgium

**Keywords:** alloimmune risk, crossmatch, high-risk transplantation, individualized immunosuppression, molecular HLA mismatch

## Abstract

Different types of kidney transplantations are performed worldwide, including biologically diverse donor/recipient combinations, which entail distinct patient/graft outcomes. Thus, proper immunological and non-immunological risk stratification should be considered, especially for patients included in interventional randomized clinical trials. This paper was prepared by a working group within the European Society for Organ Transplantation, which submitted a Broad Scientific Advice request to the European Medicines Agency (EMA) relating to clinical trial endpoints in kidney transplantation. After collaborative interactions, the EMA sent its final response in December 2020, highlighting the following: 1) transplantations performed between human leukocyte antigen (HLA)-identical donors and recipients carry significantly lower immunological risk than those from HLA-mismatched donors; 2) for the same allogeneic molecular HLA mismatch load, kidney grafts from living donors carry significantly lower immunological risk because they are better preserved and therefore less immunogenic than grafts from deceased donors; 3) single-antigen bead testing is the gold standard to establish the repertoire of serological sensitization and is used to define the presence of a recipient’s circulating donor-specific antibodies (HLA-DSA); 4) molecular HLA mismatch analysis should help to further improve organ allocation compatibility and stratify immunological risk for primary alloimmune activation, but without consensus regarding which algorithm and cut-off to use it is difficult to integrate information into clinical practice/study design; 5) further clinical validation of other immune assays, such as those measuring anti-donor cellular memory (T/B cell ELISpot assays) and non–HLA-DSA, is needed; 6) routine clinical tests that reliably measure innate immune alloreactivity are lacking.

## Introduction

Over time, donor and recipient profiles have changed substantially (1) modifying the risk of allograft rejection. Thus defining distinct alloimmune and non-alloimmune factors driving allograft rejection is greatly needed. For example, the proportion of sensitized [i.e., with circulating anti-human leukocyte antigen (HLA) antibodies] patients on kidney transplant waiting lists has gradually increased worldwide, because of both the implementation of highly sensitive immune assays to identify them and the increased proportion of retransplantations. In parallel, the number of expanded-criteria donors (ECD) or donors after circulatory death (DCD)—both groups that are often dominated by elderly people—may now exceed 50% in many transplant programmes. In such scenarios, it can be difficult to attain the excellent kidney transplantation outcomes observed for low-risk recipients and standard-criteria donors. However, low-risk donor and recipient is the usual pairing included in randomized controlled trials investigating new molecules and immunosuppression strategies. Given their real-world complexities, it would be useful to establish endpoints to identify clinically relevant and affordable improvements in outcome for distinct high-risk transplantation scenarios. This article presents evidence-based key determinants in immunological and non-immunological risk stratification, including but also extending beyond clinical research settings.

Technologies to assess alloimmune risk in transplant recipients have been developed and implemented in clinical practice, but further improvements to alloimmune risk stratification in kidney transplantation are needed. Such improvements would help to identify different subgroups of transplantation patients with distinct immune risks, which in turn would inform the development of clinical studies. Risk stratification is an essential first step toward personalized immunosuppression strategies for kidney transplant recipients.

Long-term immunosuppressive therapy may cause transplant recipients to experience various clinical events including cardiovascular disease, oncologic or metabolic complications, or opportunistic infections. Currently it is hard to individualize immunosuppressive therapy regimens to minimize the risk of such complications since data on individualization strategies remain limited and do not yet enable specific high-risk profiles to be identified (2). By contrast, the risk of allograft rejection (i.e., the immune-mediated destruction of transplanted organs), a major cause of graft loss, has been extensively investigated in large, retrospective population-based cohorts (3,4).

## Strategies to Evaluate Alloimmune Risk

Alloantigens are unavoidably recognized by the kidney transplant recipient’s adaptive immune system. However, the innate immune system—which is triggered by damage-associated molecular patterns (DAMP) released in the circulation, because of ischemia-reperfusion injury (IRI) immediately after transplantation—is necessary to prime the adaptive alloimmune response. DAMP are strong stimulators of the immune system (5,6). Immunological dogma holds that rejection requires effectors of the adaptive immune system, namely alloreactive cytotoxic T cells and donor-reactive B cells, which produce destructive donor-specific antibodies (DSA). Notably, a key feature of the adaptive immune system compared with the innate immune system is that the former generates antigen-specific memory effectors (i.e., memory T and B cells), which respond rapidly when the same antigen is re-encountered. Importantly, although this vision of rejection as being largely dependent on the ability of the adaptive immune system to discriminate between alloantigens (i.e., a process named allorecognition) largely remains dominant, independent reports from basic-research and early clinical studies suggest that some innate effectors (including monocytes and natural killer cells) are also capable of allorecognition, leading to previously overlooked types of “innate” rejection episodes (7–9) and interfering with the adaptive immune mechanisms at stakes in “classical” rejection episodes (10).

Two main strategies are used worldwide for immune-risk stratification before kidney transplantation (11). First, evaluation of HLA disparity between recipient and donor, which quantifies the risk that a “naïve” transplant candidate will develop a *de novo* alloimmune response over time, by recognizing foreign alloantigens. Secondly, identification of preformed circulating IgG antibodies against HLA in the recipient’s serum, capable of lysing donor lymphocytes in a complement-dependent manner (“serological memory”); these antibodies are identified using a complement-dependent cell (CDC)-crossmatch assay. The latter approach aims to identify sensitized transplant candidates with *preformed* humoral alloimmunity, able to trigger complement cascade activation against the graft (i.e., preformed DSA responsible for rapid severe AMR and graft loss).

Advances in the characterization of donor/recipient HLA disparities at the molecular level, use of the flow crossmatch (FCXM) (in some centers), and novel and highly sensitive immunological tools to detect circulating IgG anti-HLA antibodies (whether complement binding or not), have substantially changed the landscape of immune-risk profiling.

## Immunogenicity of Kidney Graft Depends on Donor Characteristics

Kidney transplants from donors who are elderly, ECD, ECD/DCD, or kidneys with pre-existing lesions have poorer prognoses than transplants from standard-criteria donors. Recipients of organs from high-risk donors tend to have poor renal function, with reduced medium-term graft survival (12,13). Such patients are also at high risk of delayed graft function (DGF) (14,15). These problems are not only related to the lower intrinsic quality of such organs but also to their highest level of immunogenicity (discussed more, below).

Immunosuppressive regimens administered to recipients of kidneys from ECD are adapted to avoid early acute rejection, which might worsen any pre-existing or ischemic injury of the graft; conversely, the goal of maintenance immunosuppression in such settings is to attenuate the long-term nephrotoxicity of calcineurin inhibitors. Indeed, reducing IRI in high-risk donors has been a major goal in kidney transplantation to minimize not only the risk of DGF but also to abrogate subsequent alloimmune activation favoring allograft rejection (16). Agents counteracting the effects of ischemia have been studied in selected populations, mainly by using donor/recipient risk indices to assess DGF risk (17). Interventional studies to prevent graft IRI have generally evaluated DGF occurrence as a qualitative phenomenon, although some also evaluated medium-term renal function. Given that IRI is a dynamic response to numerous molecular events, assessing DGF severity could help with the quantitative evaluation of the protective effects of anti-ischemic agents. Indeed, following discussions with the US Food and Drug Administration, this approach is in clinical investigation (ClinicialTrials.gov identifier: NCT02610296), with DGF severity (measured in terms of the number of dialysis sessions required in the first 30 days post transplantation, for participants starting dialysis on days 0–7) as the primary endpoint. However, DGF is unspecific and only partially relates to long-term graft function.

It is beyond the scope of the present paper to discuss ECD criteria and the definition of DGF as a potential endpoint in more detail, although it may also be a consequence of an early acute rejection episode; instead, we focus on the establishment of alloimmune risk in transplantation settings. Nevertheless, it should be noted that we consider DGF as a potentially meaningful endpoint for registration trials in transplantation.

## Optimizing Outcomes for Highly Sensitized Patients

Compared with other candidates, highly sensitized patients have reduced access to kidney transplantation and worse allograft outcomes, mainly due to their high risk for antibody-mediated rejection (AMR) (18).

The broadness of anti-HLA sensitization is evaluated using calculated panel-reactive antibody (cPRA) testing, which for each candidate estimate the percentage of donors against whom he is likely to show DSA, thus ultimately determining the proportion of unacceptable donors for a given transplant candidate. Candidates with very high cPRA values (>90%) have a reduced chance of finding a suitable kidney donor (19).

Several strategies enhance access to transplantation in highly sensitized candidates. The best option is the transplantation of a kidney from an HLA-compatible living donor (20,21) which, in the absence of an HLA-identical sibling volunteering for donation, may be achieved through large paired-donor exchange pools.

In the absence of a compatible donor, in the United States, desensitization protocols are commonly used (22). Standard-of-care desensitization regimens are based on a combination of off-label agent usage and techniques that aim to reduce antibody titers transiently, such as administration of intravenous immunoglobulin (IVIg), rituximab, and pre- and/or post-transplant apheresis with plasma exchange or immunoadsorption. However, such approaches are not widely followed in Europe, for reasons including evidence of inferior outcomes compared with HLA-compatible transplantation and a lack of robust data demonstrating the superiority of these high risk costly procedures (20,23,24).

In the absence of an HLA-compatible living donor, three strategies exist to increase access to transplantation for highly sensitized candidates on deceased-donor transplant waiting lists in Europe, which consider the degree of sensitization in algorithms for organ allocation. First, in the United States and some European countries including Spain and France, Kidney Allocation Systems prioritize candidates with very high cPRA values (percentages differ among countries but are ≥95%). This has increased the transplantation rate among highly sensitized candidates to levels similar to those for other candidates; however, in extremely sensitized patients (cPRA ≥99.9%), transplantation rates remain significantly lower than rates for less-sensitized patients (25). Secondly, the Eurotransplant International Foundation developed the Acceptable Mismatch (AM) Program for highly sensitized patients in the 1980s. Between 1989 and 2017, over 2,500 patients were listed on the Program, 57% of whom received a donor kidney (26). The 10-years graft survival rate among recipients listed on the Eurotransplant AM program was comparable to that for less sensitized recipients (26). The AM strategy is also used outside the Eurotransplant Program. For example, since 2005 France has operated a national AM policy (27). The EUROpe-wide Strategy to enhance Transplantation of highly sensitized patients based on Acceptable HLA Mismatches (EUROSTAM) project has developed and tested a tool to evaluate opportunities for sharing kidneys across different countries; the aim of this initiative is to increase HLA-compatible transplantation rates and thus, improve outcomes (28). There is also a third option: desensitization can be undertaken, sometimes in combination with specific allocation programs, to facilitate transplantation in sensitized recipients with preformed DSA (and/or positive crossmatch) (29,30).

Despite the creation of these programs to increase access to transplantation for highly sensitized candidates, a substantial number of people may not benefit (31), especially those with cPRA ≥98%, who often remain wait-listed for many years. These transplant candidates may need different strategies to increase their level of access to organs. In this regard, access to transplantation might be considered as a discrete endpoint among highly sensitized candidates enrolled in studies investigating whether new therapeutic approaches help to improve transplantation rates. Of note, fair evaluation of desensitization strategies based on access to transplantation requires that future studies enrol patients with homogeneous humoral immunological risk (discussed below). Clear distinction appears to be mandatory between candidates with positive lymphocytotoxicity test (LCT) cross match against their donor (who require therapeutic action pre-transplantation to reduce the titer of preformed DSA, to prevent hyperacute rejection) and candidates with lower DSA levels (positive FCXM and/or positive solid phase assay) and negative LCT, who can be transplanted without prior desensitization and only require adaptation of immunosuppression. In this regard, the transplantation rate alone is not a sufficient endpoint; only successful (e.g., rejection-free, good renal function) transplantations in the medium- or long-term (typically >10 years) should be considered. Highly sensitized transplant recipients are at high risk of developing AMR; they also have poor renal function and low graft survival rates (20,32). Although the relevance and impact of T cell-mediated rejection in these patients is lower compared with other transplantation groups, AMR with donor-specific anti-HLA antibodies exerts a detrimental effect on long-term graft survival (33,34). Hence, AMR could be a very suitable primary endpoint and surrogate for graft outcome in highly sensitized compared with HLA-compatible kidney transplant recipients.

The 2017 Banff conference described active AMR—which has several clinicopathological subtypes—as being indicative of ongoing disease activity. Active AMR is characterized by microvascular inflammation with or without graft remodeling; it is discussed further in the article by Becker et al. in the present Special Issue (35), and in Banff consensus publications (36,37).

## Considerations to Improve Stratification of Alloimmune Risk Associated With Kidney Transplantation

### Immunological Profiling of the Graft

As mentioned earlier, the immune system does not mount a response against a protein antigen without an adjuvant, which provides the molecular signals necessary to prime immune-effector cells. In transplantation, several epidemiological studies report that kidneys from older or marginal donors (i.e., those with heightened levels of tissue inflammation) are more immunogenic than kidneys from donors with less inflammation—especially when given to young recipients, whose immune system is more responsive to simulation. For instance, IRI can be increased by factors such as DCD and long cold-ischemia time, and can lead to DAMP release (36,37), thus instigating alloimmune responses. There is no validated clinical tool to evaluate the confounding effect of the type of transplantation. However, we believe that experimental data clearly support the notion that transplantations performed with living-donor kidneys carry significantly lower immunological risk compared with transplantations performed with kidneys from DCD with similar antigenic load (38).

### Immunological Compatibility Between Donors and Recipients

The risk of the recipient’s immune system developing a response against the donor kidney (allograft immunogenicity) depends on the number of potential antigenic targets, and the level of stimulation of the recipient’s immune system by adjuvant molecules.

Large studies show that long-term kidney graft survival decreases with the number of HLA-mismatch antigens between donor and recipient (39,40). HLA mismatches used to be defined based on serological determination of A, B, and DR molecules in donor and recipient. Immunogenetic advances have improved the accuracy of donor/recipient HLA typing and revealed that not all HLA mismatches have the same impact on outcome. The immunological importance of a given HLA mismatch depends on the number of epitopes that can be recognized by the recipient’s immune system (B and/or T cells) (41,42).

Progress in bioinformatics has facilitated integration of all these data to calculate the “epitope load”—a parameter that correlates much better with risk of developing dnDSA than simply counting the number of HLA mismatches (43–46). Epitope load is likely to play a key role in better stratifying the primary immunological risk associated with a specific transplantation and are associated with specific geographical regions that may not be extrapolated to a global level.

Furthermore, beyond mere quantity, not all epitopes appear to have the same immunogenic relevance: although qualitative aspects of epitopes are not well documented, publications have described certain physicochemical characteristics of different epitopes (47,48).

However, without consensus regarding which algorithm (and cut-off) should be used, and with the ongoing need for more comprehensive high-resolution (HR) HLA typing, it might be difficult to integrate such information immediately in clinical practice. The principle of diminishing epitope load can be implemented irrespective of a selected algorithm: using HLAMatchmaker, amino acid mismatching and physicochemical mismatch load were shown to have the same impact on outcome (49). Accordingly, we consider that in presence of complete HR donor/recipient HLA typing, or low resolution within biologically related pairs, transplantations performed with HLA-identical donors (in particular if donor and recipient are closely related, e.g., siblings) carry significantly lower immunological risk than those performed with donors of other HLA statuses.

#### Anti-HLA Antibodies

Screening for anti-HLA antibodies is the cornerstone of immune-risk profiling in kidney transplant recipients. A positive CDC-crossmatch with donor cells is considered a contraindication to transplantation (unless desensitization is initiated before transplantation, a situation that is not discussed in the present article). CDC-crossmatch can also be assessed with a panel of different cells to evaluate the diversity of the recipient’s serological memory against HLA molecules. A CDC-PRA test figure of >80% was historically used to define hyperimmunized patients and implies a lower access to transplantation (as their CDC-crossmatch with donor cells is more likely to be positive). Percentage of PRA has been applied for immune-risk stratification in large clinical trials. However, since the CDC-crossmatch only detects DSA that activate complement [a characteristic that depends on the titer and specific biological characteristics of IgG (50)], some recipients with negative CDC-crossmatch and/or CDC-PRA might still reveal preformed DSA that also have a deleterious impact on graft survival through antibody-dependent cell cytotoxicity (21,51–53).

While a negative CDC-crossmatch with donor cells will remain a mandatory condition to perform transplantation, novel, or more sensitive techniques—such as FCXM and single-antigen bead (SAB) assays to detect alloantibodies—have been implemented to improve the screening of recipients for preformed DSA. These assays can detect circulating anti-HLA antibodies that can be a mixture of antibodies that do and do not fix complement and may harm the graft through antibody-dependent cell cytotoxicity and/or direct modulation of graft endothelial-cell biology (54), thus significantly improving the capacity of detecting pathogenic circulating DSAs. FCXM with donor cells is more sensitive than CDC and yields fewer false-positive results than solid-phase assay (55–57). However, it requires collection of the donor’s cells and use of a cytometer, which is not available in all immunogenetic laboratories. Other limitations of FCXM include poor standardization, thresholds, and interpretation of test systems. Conversely, SAB assays are widely available, more standardized, and have better reproducibility than FCXM. SAB assays consist of microparticles coated with purified HLA antigens; if antibodies are present, a semiquantitative readout is provided. There are two commercial platforms: One Lambda^®^ (Thermo Fisher Scientific, Canoga Park, CA, United States) and Immucor^®^ (Immucor, Norcross, GA, United States) with rather good correlation and reliability between both assays (58). With some caveats, we propose that SAB assays should be the gold standard to establish the repertoire of serologic memory and define the presence of a recipient’s circulating DSA. The caveats are that the results of SAB assays are semiquantitative, and they have certain technical limitations and interlaboratory variability: for example, it is necessary to prevent the artifact of complement interference by pre-treating serum (e.g., with EDTA or heat inactivation). In the absence of strong consensus to define the mean fluorescence intensity (MFI) cut-off that would indicate clinically relevant HLA antibodies, we suggest transplant physicians and immunologists should define the most appropriate cut-off for local circumstances. Establishing plausibility of the potential DSA, considering previous immunizing events, is a key factor to determine antibody positivity in the individual. Notably, several studies have reported that the ability of DSA identified by SAB to bind *ex vivo* donor cells in FCXM is a good predictor of subsequent AMR lesions and graft loss (in 50% and 30% of recipients, respectively) (21,52,57,59–61). Together, these data suggest that optimal performance of FCXM in identifying pathogenic DSA depends on both higher specificity (elimination of false positivity due to denatured HLA molecules on SAB) and lower sensitivity (so that only DSA with high titers are detected).

**FIGURE 1:**
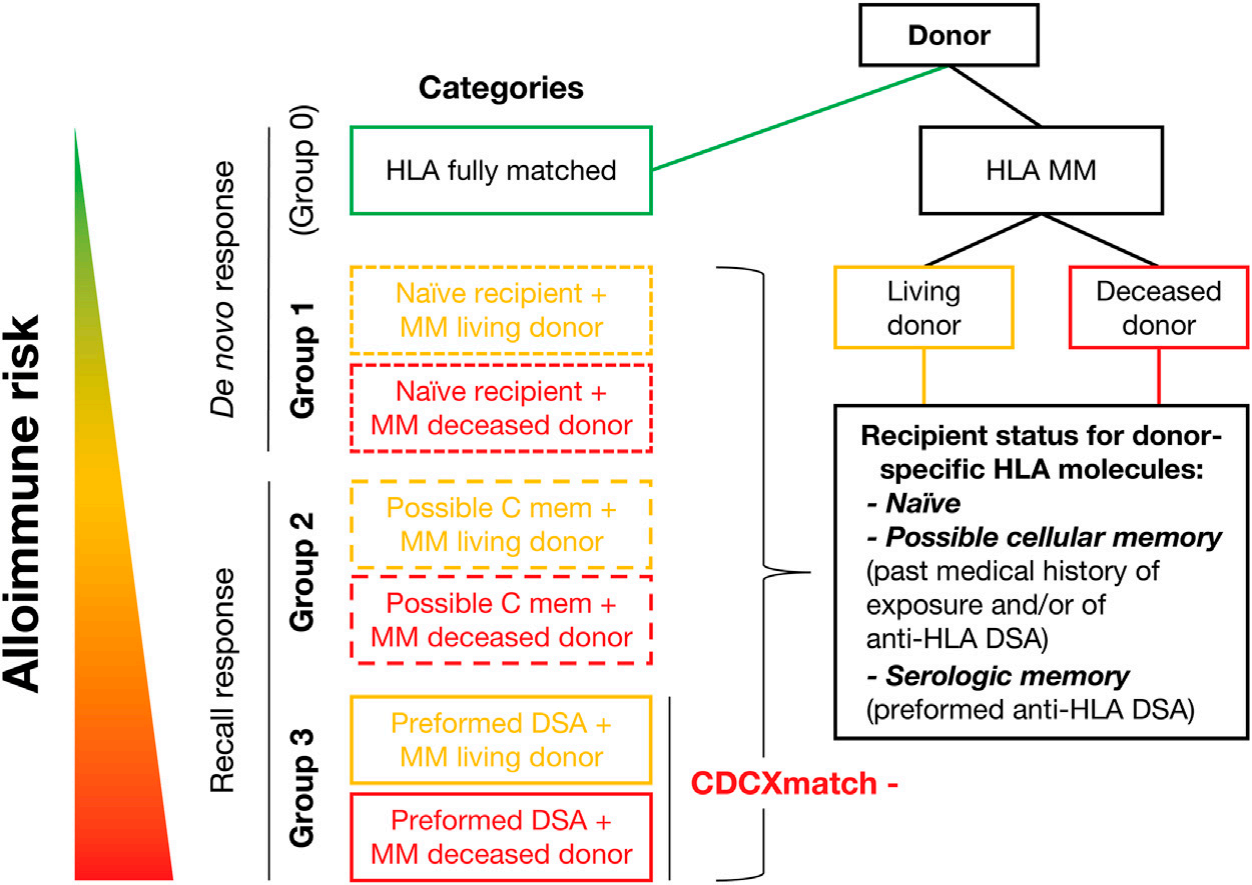
Proposed structure for stratifying alloimmune risk.

Utilizing the results of these analyses, transplant candidates could be categorized according to their level of immune sensitization at the time of transplantation. In alignment with the approach proposed by the STAR [Sensitization in Transplantation: Assessment of Risk (21,51,52)] and ENGAGE [EuropeaN Guidelines for the mAnagement of Graft rEcipients (11)] Working Groups, ESOT recommends differentiation of anti-HLA antibody status by categorizing patients (Figure 1). Using this approach, patients with HLA-DSA at the time of transplantation (day 0; group 3, Figure 1) would have a higher likelihood of post-transplant AMR and less favorable allograft outcomes than patients naïve for alloantigen (group 1, Figure 1). The situation is less clear for group 2 (patients with previous exposure to donor HLA antigens during a transplant or pregnancy or a history of HLA-DSA, but who are negative at the time of transplantation). A retrospective single-center study suggested a detrimental impact on outcome for these people (62). We recommend considering patients in group 2 as being at intermediate risk, because of the likely presence of cellular memory (discussed below) (Figure 1). There is ongoing discussion about the predictive value of the MFI in SAB testing of HLA-DSA (29) and whether the MFI could be used as a potential surrogate to estimate the HLA-DSA titer and, consequently, further refine the individual’s risk of developing AMR in group 3 patients. However, technical aspects of the semiquantitative values should be considered: cut-offs may differ between centers.

#### Non-HLA Antibodies

Not all antibodies implicated in kidney transplant rejection are directed against the HLA system. Accumulating clinical and experimental data indicate a deleterious role of antibodies, i.e., antibodies directed against graft antigens other than allogeneic HLA molecules (63,64). Of note, the nature of these antibodies, i.e., whether they are auto and/or alloreactive, remains currently unclear. In this regard, the demonstration that genetic mismatch of non-HLA haplotypes coding for transmembrane or secreted proteins is associated with an increased risk of functional graft loss, independently of HLA incompatibility, suggests that non-HLA antibodies could be alloreactive (65). Furthermore, this literature suggests there is enormous diversity among potential antigenic targets, complicating the detection of non–HLA-DSA. Until consensus is established, and in the absence of a validated assay (and cut-off value), we do not recommend that non–HLA-DSA are considered when evaluating the immunological risk for a transplantation.

#### Adaptive Cellular Memory

In addition to immunological assessment of antibodies, screening for adaptive cellular memory seems to be valuable. Although ELISpot assays can identify donor-reactive memory B and T cells in kidney transplant recipients, to date these assays have only been shown to predict transplant outcomes in small, underpowered, retrospective studies (66–68). Standardization and cross-validation of the donor-specific T cell ELISpot assay between laboratories has been performed (69,70) and translation to clinical settings was attempted in a multicenter, randomized interventional trial (71). According to this study, rates of T cell-mediated rejection were significantly higher in patients with preformed donor-reactive T cell frequencies compared with other patients. However, these cell-based assays need further evaluation of reproducibility before widespread clinical use.

Although we do not recommend implementation of T/B cell ELISpot assays in routine clinical practice, determining the presence of an adaptive cellular memory in the recipient seems important, to stratify the immunological risk of a specific transplantation according to immune sensitization status. This can be done by establishing the recipient’s pregnancy history (to identify the father’s HLA type), transplantation history (to identify the previous donor’s HLA type), and transfusion history (red blood cells and platelets, although the profile of sensitization is usually complicated to assess) (72). While we acknowledge that this information might not be obtained in many cases and does not necessarily imply the presence of an effector anti-donor alloimmune response at the cellular level, in some specific transplant scenarios (e.g., living donor kidney transplantation) this information might be possible to retrieve and may help to better understand potential immunological events occurring during the early phases post-transplantation, underscoring a preformed recall anti-donor alloimmune response. A patient with a history of anti-HLA antibodies that are undetectable in the circulation should be considered likely to have memory B and/or T cells against these HLA antigens, especially those against previously recognized alloantigens. While there is no evidence on how to specifically manage these patients, such situations require special attention.

#### Innate Immune Effectors

Finally, as well as participating in graft damage on recruitment by adaptive effectors, innate immune effectors might be able to recognize allogeneic non-self (7–9). While we wait for experimental studies to translate into robust clinical findings, and reliable assays are developed to guide decisions, we do not recommend that innate immune alloreactivity is evaluated in routine clinical practice.

## Conclusions

The following is our proposal for alloimmune risk stratification in CDC-negative kidney transplantation.

• Transplantation performed with an HLA-identical donor carries a significantly lower immunological risk than transplantation from a donor of another HLA status.
• For the same allogeneic eplet load, grafts from living donors, which are better preserved and are therefore less immunogenic than grafts from deceased donors, are associated with a lower immunological risk.
• Patients with anti-donor serological memory at the time of or short time before transplantation (i.e., those with the presence of HLA-DSA) should be clearly differentiated from the others:

 ○ Patients with donor reactivity are likely to have immune reactions to the allograft, with a heightened risk of post-transplant AMR and poor allograft outcome.
 ○ SAB testing is the gold standard to establish the repertoire of serologic memory and define the presence of a recipient’s circulating anti-HLA DSA.

  – Local transplant physicians and immunologists should determine the appropriate cut-off point, with a focus on plausibility of immunization history.
  – In absence of clinical validation, non–HLA-DSA routine screening assays should not be considered when evaluating immunological risk of a transplantation.

 ○ Using SAB testing, three risk groups can be identified (patients with non–donor-specific HLA antibodies and no previous exposure to donor antigen are considered as naïve patients):

  – Group 1: Patients with no signs of anti-HLA immune sensitization at any time point (very low risk).
  – Group 2: Patients with previous exposure to donor antigens or history of HLA-DSA positivity, but without HLA-DSA at time of transplantation (intermediate risk due to likely presence of memory T and/or B alloimmune response): T/B ELISpot assays could identify anti-donor memory cells, but without clinical validation these assays should not be considered when evaluating immunological risk of a transplantation.
  – Group 3: Patients with HLA-DSA at time of transplantation (high risk). There is ongoing discussion on the utility of MFI in SAB or FCXM as a potential surrogate to estimate the DSA titer and for individual risk stratification.
  – Molecular HLA mismatch analysis is likely to play a future role in better allocating more compatible allografts, as well as in stratifying the primary alloimmune risk. However, in the absence of consensus regarding what algorithm (and which cut-off) should be used to quantify the eplet load and whether the quality of eplet should also be considered, it is difficult to integrate such information immediately into clinical practice and clinical trial design. Further consensus building is necessary.
  – Although it is a fast-evolving field, no reliable test is currently available to measure innate immune alloreactivity in routine clinical practice.

### Scientific Advice From the Committee for Medicinal Products for Human Use (CHMP) of the European Medicines Agency (EMA) Regarding these Conclusions

• The CHMP agreed that several important issues need to be considered in assessing the alloimmune risk following kidney transplantation. These include general characteristics of the recipient and donor, as well as issues related to the transplanted organ and issues requiring further studies and/or consensus before adapting into general guidelines.
• Several of these factors and issues are already discussed in the EMA guideline CHMP/EWP/263148/06 (66). It is agreed that high-risk populations should be distinguished based on 1) greater risk of clinical events and 2) the need for different immunosuppression intensity.

 ○ Regarding the immunological risk related to the donated kidney, the CHMP agreed that the number of antigenic targets on the donated organ and “adjuvantation” affect the outcome of transplantation. Some of these issues will be addressed by the type of organ transplanted (ischemia time, HLA mismatch, living donation vs. ECD, DCD etc.), which, depending on the study design, can be used for stratification.
 ○ Regarding improving stratification of the recipients based on immunological profiling, the CHMP agreed that:

  – A positive CDC-crossmatch detects only DSA that activate complement. For risk stratification, this is not ideal, as DSA may still be present with deleterious impact on graft survival.
  – Other tests are more sensitive, such as the FCXM and SAB assays. ESOT proposes to use the SAB assay as the gold standard to define the presence of recipient’s circulating DSA. The preference of SAB is advocated for sensitive anti-HLA DSA based on wide availability in practice. No data were submitted to support this conclusion. Furthermore, no definitive metrics are proposed (e.g., MFI cut-off values), leaving the cut-off definitions to local transplant physicians and immunologists. This flexibility of defining cut-offs in clinical practice is acknowledged. However, this raises issues for external validity of study results when the proposed metrics are not generally accepted.
  – Innate immune effects and cell-based assays addressing cellular memory need further evaluation before widespread clinical use and validation before application in clinical trials.

 ○ The CHMP stated that the classification in three risk categories based on the HLA antibody profiles is interesting and could be acceptable, if a general consensus in the transplant community supports the classification. Also, the definition of cut-offs to define anti-HLA positivity requires more work. Currently, for individual applications basis.
 ○ Finally, the CHMP stated that the stratification factors to be used in individual studies should reflect the goal and the size of the study.
